# Global DNA Methylation in Children with Posterior Urethral Valves: Association with Kidney Function and Kidney Scarring

**DOI:** 10.3390/ijms27135649

**Published:** 2026-06-23

**Authors:** Sachit Anand, Anjali Srivastava, Ajay Verma, Himalaya Kumar, Jitendra Meena, Chittaranjan Behera, Kalpana Luthra

**Affiliations:** 1 Department of Pediatric Surgery, All India Institute of Medical Sciences, New Delhi 110029, India; 2 Department of Pediatrics, All India Institute of Medical Sciences, New Delhi 110029, India; 3 Department of Forensic Medicine and Toxicology, All India Institute of Medical Sciences, New Delhi 110029, India; 4 Department of Biochemistry, All India Institute of Medical Sciences, New Delhi 110029, India

**Keywords:** epigenetics, DNA methylation, posterior urethral valves, chronic kidney disease, congenital anomalies of the kidney and urinary tract

## Abstract

Posterior urethral valves (PUV) cause congenital urinary tract obstruction and often lead to chronic kidney disease (CKD) despite treatment; however, DNA methylation remains underexplored in PUV. This cross-sectional study aimed to compare peripheral-blood global DNA methylation, measured as 5-methylcytosine content (5-mC%), between boys with PUV and age-matched male controls, and to assess its association with kidney function, CKD stage, and kidney scarring. The study included 45 boys with PUV and 45 age-matched boys as controls. Peripheral-blood global 5-mC% was quantified using an ELISA-based assay. Glomerular filtration rate and kidney scarring were assessed by nuclear scintigraphy, and PUV patients were categorized according to CKD stage and scarring status. Statistical comparisons were performed using the Kruskal–Wallis test followed by Dunn’s test, with exploratory trend analysis used to evaluate the association between global 5-mC% and CKD severity. Global 5-mC content was significantly higher in PUV patients than in controls (median 5-mC%: 0.4336 vs. 0.3732, *p* < 0.001). Within the PUV cohort, global 5-mC% increased with CKD severity (*p* < 0.05) and showed a logarithmic association with CKD stage (R^2^ = 0.8012). Patients with kidney scarring also had significantly higher global 5-mC% than controls (*p* < 0.001), although differences across individual CKD stages and scarring subgroups were not statistically significant. These findings suggest altered systemic global 5-mC content in boys with PUV and support larger, longitudinal studies incorporating locus-specific methylation profiling.

## 1. Introduction

Chronic kidney disease (CKD) in the pediatric population is a significant global health concern, with congenital anomalies of the kidney and urinary tract (CAKUT) being the leading cause of pediatric kidney failure [1]. Among these anomalies, posterior urethral valves (PUV), a congenital malformation of the male urinary tract, represent one of the most common causes of CKD in male infants, affecting approximately 1 in 5000 live male births [2].

The early detection and monitoring of CKD severity remain challenging in PUV due to the variability in disease presentation and the lack of sensitive biomarkers that can accurately track impairment of kidney function, particularly in the early stages when therapeutic interventions could be most effective [2,3]. Many children with PUV experience significant kidney damage by the time their disease is diagnosed. In addition, a substantial proportion of these children experience progressive deterioration in kidney function despite successful valve ablation.

The long-term kidney outcome in PUV is influenced by multiple factors, including the severity and timing of urinary tract obstruction, bladder dysfunction, recurrent urinary tract infections (UTIs), kidney dysplasia, and progressive tubulointerstitial injury [2]. Even after adequate surgical relief of obstruction, ongoing bladder dysfunction and established kidney injury may contribute to continued deterioration of kidney function [2]. Therefore, identifying the molecular changes associated with kidney dysfunction in PUV may help improve understanding of disease progression and support the development of biomarkers for risk stratification and longitudinal monitoring.

Recent advancements in molecular biology have highlighted the critical role of epigenetic modifications, particularly DNA methylation, in regulating gene expression and cellular function. DNA methylation, a well-known epigenetic mechanism, has been implicated in the pathogenesis of various diseases, including kidney diseases, where it may influence inflammatory, fibrotic, and metabolic pathways relevant to kidney dysfunction [4,5,6]. Global DNA methylation quantification in peripheral blood has been applied in CKD cohorts, supporting the feasibility of global methylation assessment in kidney disease [7,8]. However, its role in congenital uropathies such as PUV has been largely unexplored. In this context, DNA methylation may hold promise as a potential diagnostic and prognostic marker, providing insights into the molecular mechanisms associated with disease severity and enabling earlier detection or monitoring of kidney dysfunction.

As a first step in an epigenetically unexplored condition, quantifying global 5-mC content provides a feasible screening measure to establish whether a methylation signal is present, thereby informing subsequent locus-specific and longitudinal studies. Thus, the primary objective of this study was to compare peripheral blood global DNA methylation (5-mC%) between boys with PUV and age-matched boys as controls. The secondary objectives were to evaluate the relationship between methylation and kidney function (GFR and CKD stage), and to explore differences by kidney scarring status. By comparing global DNA methylation levels between cases and controls and among patients with varying disease severity, we provide preliminary data on DNA methylation in PUV and a rationale for future mechanistic studies. To the best of our knowledge, this study is the first to evaluate global DNA methylation in boys with PUV.

## 2. Results

### 2.1. Patient Characteristics

The control group (n = 45) had a median age of 61 months (IQR = 32.5–114), while the PUV group (n = 45) had a median age of 33 months (IQR = 12–108). No significant difference was observed in age distribution between the two groups (*p* = 0.20). Controls had no comorbidities and were not receiving any medications at the time of sampling. In the PUV cohort, 6/45 (13.3%) patients were receiving antihypertensive therapy, while 22/45 (48.9%) and 13/45 (28.9%) were receiving oxybutynin and prazosin, respectively (Table 1). No participant had an active UTI at the time of sampling.

GFR and DMSA reports were available for 43/45 patients. The median GFR in the PUV cohort was 70 mL/min/1.73 m^2^ (range: 13–120 mL/min/1.73 m^2^). Of the PUV patients, 8 (18.6%) were classified as Stage 1 CKD, 19 (44.2%) as Stage 2 CKD, 7 (16.2%) as Stage 3 CKD, 6 (14.0%) as Stage 4 CKD, and 3 (7.0%) as Stage 5 CKD. Similarly, 27 (62.8%) patients had no kidney scarring, 7 (16.3%) had unilateral kidney scars, and 9 (20.9%) had bilateral kidney scars (Table 1).

### 2.2. Global DNA Methylation Levels in PUV Compared with Controls

**Figure 1:**
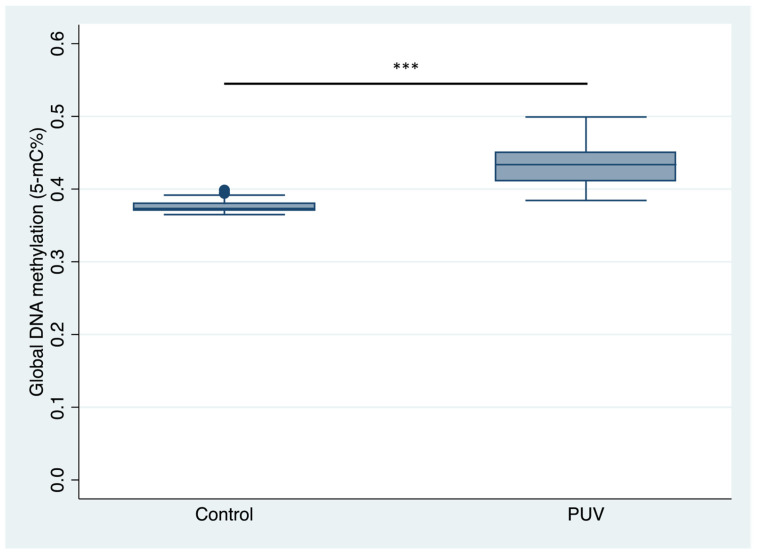
Increased global DNA methylation in PUV patients. Box plot showing significantly higher global DNA methylation (5-mC%) in PUV patients (n = 45) compared with controls (n = 45). Data are presented as the median with interquartile range. Whiskers represent adjacent values, and dots indicate outside values according to the standard 1.5× IQR rule. No data points were excluded from the analysis. Mann–Whitney U test was used to analyze the data. Statistical significance: *** *p* < 0.001.

The analysis of global DNA methylation levels (5-mC%) revealed significantly higher levels in patients with PUV compared with controls (Figure 1). The control group had a median 5-mC% of 0.3732 (IQR: 0.3700–0.3792), whereas the PUV group had a markedly higher median 5-mC% of 0.4336 (IQR: 0.4114–0.4500), corresponding to an absolute median difference of 0.0604 5-mC% units. This difference was statistically significant (*p* < 0.001, Mann–Whitney U test). Cliff’s Delta was 0.986, indicating strong distributional separation, meaning that there was a very high probability that a randomly selected PUV value would exceed a randomly selected control value.

### 2.3. Global DNA Methylation Levels Across CKD Stages

**Figure 2:**
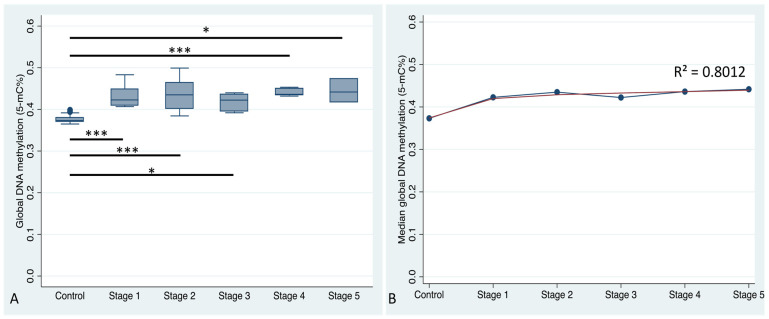
Global DNA methylation across chronic kidney disease (CKD) stages in PUV patients. (**A**) Box plot showing global DNA methylation across CKD stages (Stages 1–5) compared with controls. Boxes represent the interquartile range, with the central line indicating the median. Whiskers represent adjacent values, and dots indicate outside values according to the standard 1.5× IQR rule. No data points were excluded from the analysis. Kruskal–Wallis test followed by Dunn’s multiple comparisons test was used to analyze the data shown in panel A. Statistical significance: * *p* < 0.05, *** *p* < 0.001. (**B**) Line chart demonstrating an increasing trend in median 5-mC% across CKD stages. The blue line represents observed median 5-mC% values, and the red line represents the logarithmic fit (R^2^ = 0.8012). Distribution of participants in each group: controls (n = 45), Stage 1 (n = 8), Stage 2 (n = 19), Stage 3 (n = 7), Stage 4 (n = 6) and Stage 5 (n = 3).

A subgroup analysis of 43 PUV patients with available GFR reports provided further insights into methylation patterns. We analyzed 5-mC% across six participant groups (control and CKD Stages 1–5) using the Kruskal–Wallis test, which revealed a significant difference in the median values (*p* < 0.0001, Kruskal–Wallis statistic = 64.52). Subsequent post hoc analysis with Dunn’s multiple comparison test demonstrated a significantly elevated 5-mC% in all CKD stages compared with the control group (Figure 2A). Specifically, the differences in rank sums were as follows: Stage 1 CKD vs. control (*p* < 0.001), Stage 2 CKD vs. control (*p* < 0.001), Stage 3 CKD vs. control (*p* < 0.05), Stage 4 CKD vs. control (*p* < 0.001), and Stage 5 CKD vs. control (*p* < 0.05). However, no significant differences in the rank sums were observed among the CKD stage subgroups.

Trend analysis revealed that 5-mC% levels showed an overall increase with CKD stage. A logarithmic trendline was applied to the median 5-mC% values, as this model best captured the non-linear pattern of increasing methylation across CKD stages, suggesting a steep rise in early stages followed by a plateau (Figure 2B). This approach was also supported by the highest coefficient of determination (R^2^ = 0.8012) among the models tested. This trend suggested a possible association between global DNA methylation levels and CKD stage in PUV patients.

### 2.4. Association of Global DNA Methylation in PUV and Kidney Scarring

**Figure 3:**
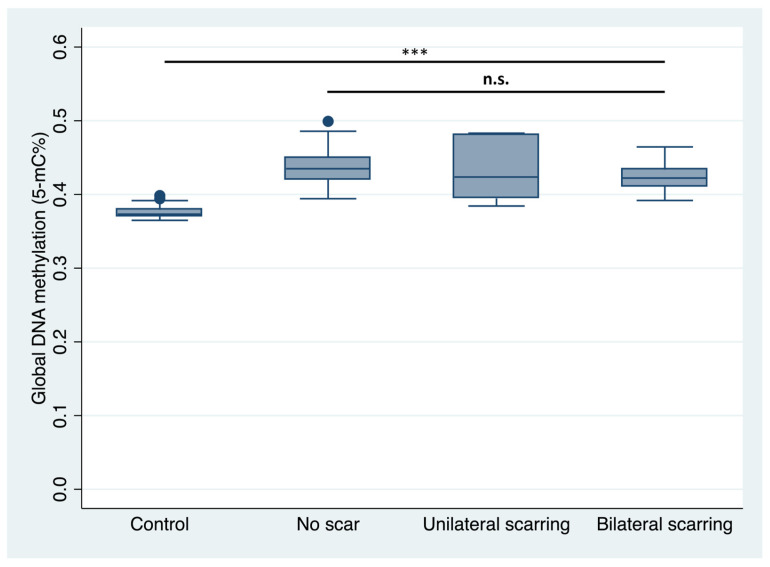
Global DNA methylation levels and kidney scarring in PUV patients. Box plot showing global DNA methylation (5-mC%) across controls and three kidney scarring categories: no scar, unilateral scarring, and bilateral scarring in PUV patients. Data are presented as the median with interquartile range. Whiskers represent adjacent values, and dots indicate outside values according to the standard 1.5× IQR rule. No data points were excluded from the analysis. Kruskal–Wallis test followed by Dunn’s multiple comparisons was used to analyze the data. Distribution of participants in each group: controls (n = 45), No scar (n = 27), Unilateral scarring (n = 7), and Bilateral scarring (n = 9). Statistical significance: *** = *p* < 0.001, n.s. = not significant.

We also assessed the relationship between global DNA methylation and the presence of kidney scarring on DMSA scans in PUV patients. The Kruskal–Wallis test was performed to compare 5-mC% across four groups: controls, no scar, unilateral scarring, and bilateral scarring. The results demonstrated significant differences in global DNA methylation levels among the groups (*p* < 0.001, Kruskal–Wallis statistic = 63.85). Dunn’s multiple comparisons test revealed significant differences between the control group and all kidney scarring groups (*p* < 0.001). However, no significant differences were observed among the kidney scarring groups (Figure 3).

## 3. Discussion

DNA methylation is a key epigenetic modification in which methyl groups are added to cytosine residues to form 5-mC, thereby influencing chromatin structure and gene regulation [9]. In many contexts, increased methylation at gene promoters or regulatory regions is associated with reduced transcription, although the functional effect is locus- and context-dependent [9,10]. Epigenetic marks are mitotically heritable and maintained across cell divisions, can be shaped by environmental and disease-related exposures, and are increasingly recognized as dynamic and potentially reversible over time [11,12,13].

This study provides preliminary evidence of altered global DNA methylation in boys with PUV compared with controls. To our knowledge, this is the first report to demonstrate higher peripheral-blood global 5-mC% in patients with PUV and to examine its association with disease severity. Notably, ELISA-based quantification of global 5-mC in whole blood has previously been reported in CKD/dialysis cohorts, supporting the feasibility of this approach in kidney disease [7,8]. Our findings are consistent with observations in other kidney diseases, including diabetic nephropathy, IgA nephropathy, and polycystic kidney disease, where DNA methylation changes, often at specific genomic loci, have been associated with inflammatory and fibrotic pathways and reduced kidney function [14,15,16]. In this context, the higher global methylation observed in PUV may represent an epigenetic signature associated with CKD status or burden in this population.

Although we quantified global rather than locus-specific methylation, these broader changes could reflect systemic epigenetic alterations accompanying CKD, such as chronic inflammation, oxidative stress, and the accumulation of uremic toxins, which are well recognized in CKD pathophysiology. These systemic changes may parallel biological processes relevant to kidney injury (including fibrosis, immune activation, and tubular stress) and support the rationale for future studies using higher-resolution methylome approaches to determine locus-specific patterns and their relationship to kidney phenotypes in PUV.

Prior studies in CKD have reported heterogeneous DNA methylation findings. Some studies have described global methylation abnormalities, including hypomethylation in uremic or CKD-related states, whereas genome-wide and locus-specific studies have shown that both hypermethylation and hypomethylation may occur at different genomic loci in CKD [17,18,19]. These apparently divergent findings may reflect differences in disease etiology, tissue or biospecimen type, assay platform, genomic resolution, CKD stage, metabolic status, and treatment exposure [20]. Therefore, the increased peripheral-blood global 5-mC% observed in the present PUV cohort should not be interpreted as uniform hypermethylation across the genome or at specific genes. Rather, it represents a broad systemic methylation signal that requires validation and further characterization using candidate gene or genome-wide approaches, such as methylation arrays or bisulfite sequencing, in larger longitudinal cohorts.

The altered methylation levels observed in PUV patients, along with the upward trend in methylation levels across CKD stages in PUV, are biologically suggestive; however, differences between individual CKD stages were not statistically significant. Global 5-mC% does not localize methylation changes to specific genes, but metabolic disturbances related to PUV or PUV-associated CKD could plausibly alter genome-wide methylation capacity by affecting methyl donor availability and the enzymatic machinery regulating 5-mC, thereby contributing to broad shifts in global methylation [21,22,23]. These mechanisms remain speculative in our dataset and require locus-specific methylome and functional correlation studies in the future.

A noteworthy finding in our study is the lack of association between global DNA methylation levels and the severity of kidney scarring. Although global DNA methylation levels in all three PUV subgroups, namely no scarring, unilateral scarring, and bilateral scarring, differed significantly from those in the control group, no significant difference was observed when the methylation levels were compared among the scarring groups. The exact reason for this finding remains unclear; however, kidney scarring involves a complex interplay of inflammatory, immune, and fibrotic pathways [24]. This complex process may also involve genetic, epigenetic, and environmental factors and may not be captured by global methylation measures alone.

Another potential reason for the absence of significant differences in DNA methylation levels among the CKD groups or among the kidney scarring groups is the limited sample size, which may have reduced the statistical power to detect subtle variations. Expanding the cohort size could address this limitation and improve the robustness of the findings. Furthermore, global methylation analyses using higher-resolution techniques, such as bisulfite sequencing or methylation arrays, may allow for the identification of specific methylation patterns associated with kidney scarring severity.

One critical challenge in managing CKD is the timely detection and longitudinal monitoring of disease severity, particularly during the transition from Stage 3 CKD to kidney failure, when GFR drops below critical thresholds [25]. Our findings suggest that peripheral-blood global DNA methylation may represent a disease-associated molecular signal in boys with PUV; however, its role as a biomarker for monitoring disease progression or predicting recovery requires validation in longitudinal studies with serial methylation assessment. In urological diseases, including bladder pathologies, DNA methylation signatures have been explored as non-invasive biomarkers, particularly in urine-based assays [26]. Although PUV differ biologically and clinically from bladder cancer, such studies support the broader feasibility of evaluating methylation-based markers in urological disease contexts. In the current study, methylation was assessed in peripheral blood because kidney biopsy is invasive and not ethically justifiable in children with PUV without a clinical indication. Additionally, blood-based methylation has been used in kidney disease research and has been associated with kidney function in prior studies [7,17,27,28]. However, future studies should also validate these findings using urine biospecimens, such as urine-derived kidney cells or extracellular vesicles, and kidney tissue, when clinically indicated.

Bladder dysfunction is an important component of PUV pathophysiology and may persist even after successful valve ablation, manifesting as detrusor overactivity, poor compliance, and valve bladder syndrome, potentially contributing to ongoing upper-tract stress and adverse kidney outcomes [29,30,31]. Because our study did not include a comparator group with bladder dysfunction but preserved kidney function, we cannot determine whether the observed methylation differences are driven predominantly by impairment of kidney function, bladder dysfunction, or their combination. Therefore, these findings should be interpreted cautiously as a composite, disease-associated epigenetic signal in PUV.

Factors such as diet and drug exposure should be considered when interpreting global methylation signals in PUV [32,33]. In our cohort, a substantial proportion of children were receiving medications commonly used in PUV management, including oxybutynin (48.9%), prazosin (28.9%), and antihypertensives (13.3%), which could influence methylation directly or indirectly through metabolic and hormonal pathways. Dietary components, particularly those involved in one-carbon metabolism, including folate, vitamin B12, and methionine, are well-established modulators of DNA methylation and could also play a role in the observed findings [34]. Future prospective studies should systematically capture detailed medication exposure (dose/duration) and dietary history to assess their independent association with 5-mC%.

There are some limitations and challenges associated with the current study. First, this study had a limited sample size. Small subgroup sizes after stratification by CKD stage and scarring status resulted in limited statistical power. In addition, the study did not include a non-PUV CKD comparator group, which limits our ability to determine whether the observed methylation changes are specific to PUV-related CKD or reflect CKD more broadly. Second, the findings did not translate into significant CKD stage-wise or scarring subgroup differences, likely due to small subgroup sample sizes and inter-individual variability. In addition, CKD stage and kidney scarring are correlated, and the modest sample size precluded multivariable modeling to study their independent associations with 5-mC%. Third, the cross-sectional design precludes any inference regarding causality or temporality. Age at valve ablation and the interval between valve ablation and methylation sampling were not available for all PUV patients; therefore, CKD-stage-related methylation trends should be interpreted cautiously, as they may partly reflect disease duration. Longitudinal studies in other CKD etiologies have demonstrated that DNA methylation is dynamic and may evolve with disease course [35,36], suggesting the need for future follow-up investigations in PUV to clarify these relationships. Fourth, potential confounders such as medication exposure, including oxybutynin, alpha blockers, antihypertensives, and dietary factors, including folate and vitamin B12, were not systematically controlled for in this study. Finally, the current study quantifies global DNA methylation and does not provide locus-specific insights. Future prospective, multicentric studies with larger sample sizes and high-resolution methylome profiling will be required to clarify the biological basis of these observations.

Nevertheless, this study demonstrates for the first time that global DNA methylation is significantly elevated in pediatric patients with PUV compared with controls. Importantly, methylation levels show a trend toward higher values across CKD stages, suggesting an association between altered methylation and kidney dysfunction in this cohort. Although the study has some limitations, it lays the groundwork for future mechanistic and longitudinal investigations.

## 4. Materials and Methods

### 4.1. Study Design and Setting

This single-center, cross-sectional study was conducted between June 2024 and December 2025 in the Department of Pediatric Surgery at the All India Institute of Medical Sciences (AIIMS), New Delhi, India. Ethical approval was obtained from the Institutional Review Board (Ref. No: AIIMSA1432/07.06.2024, RP-5/2024; dated 18 June 2024), and the study adhered to institutional guidelines for ethical research. Written informed consent was obtained from the parents of all children, with assent provided by participants where age-appropriate.

### 4.2. Study Population

This study included 90 male participants: 45 consecutive cases with PUV and 45 age-matched boys as controls. During the study period, all consecutively presenting eligible PUV patients were approached, and 45/47 families consented to participate. The diagnosis of PUV was based on standard clinical and radiological criteria [37]. All children with PUV had undergone successful valve ablation and were under regular follow-up at the time of recruitment.

During recruitment, only children with no clinical or laboratory evidence of active UTI were included. Clinical assessment included screening for fever, dysuria, increased urinary frequency, urgency, lower abdominal or suprapubic pain, flank pain, foul-smelling urine, vomiting, poor feeding, and lethargy, particularly in infants and young children. Physical examination included assessment of body temperature, general condition, hydration status, abdominal or suprapubic tenderness, and costovertebral angle tenderness. Laboratory screening included urinalysis for pyuria and bacteriuria, and urine culture. The UTI screening data at the time of sampling are summarized in Supplementary Table S1.

The control group comprised male children recruited through the outpatient department who presented with non-genitourinary, non-inflammatory conditions unrelated to systemic illness, such as constipation, hernia, or abdominal pain, or healthy boys attending routine vaccination. Controls were eligible only if they had no known congenital or acquired kidney disease, no chronic systemic illness, and no previous history of UTI. At the time of sampling, controls were also screened clinically and by urinalysis and urine culture to exclude active UTIs (Supplementary Table S1).

### 4.3. Assessment of Kidney Function and CKD Stage

To explore the relationship between DNA methylation and kidney function, children within the PUV group were divided into subgroups according to CKD stage. Kidney function was assessed using glomerular filtration rate (GFR) values obtained from radionuclide-based diethylenetriamine pentaacetic acid (DTPA) scintigraphy. GFR values were normalized to body surface area and expressed as mL/min/1.73 m^2^. CKD staging was performed according to the Kidney Disease: Improving Global Outcomes (KDIGO) guidelines [38]. Patients were classified as follows: Stage 1 CKD (mild kidney dysfunction, GFR ≥ 90 mL/min/1.73 m^2^); Stage 2 CKD (mild to moderate kidney dysfunction, GFR 60–89 mL/min/1.73 m^2^); Stage 3 CKD (moderate to severe kidney dysfunction, GFR 30–59 mL/min/1.73 m^2^); Stage 4 CKD (severe kidney dysfunction, GFR 15–29 mL/min/1.73 m^2^); and Stage 5 CKD (end-stage kidney disease, GFR < 15 mL/min/1.73 m^2^ or patients requiring dialysis).

### 4.4. Assessment of Kidney Scarring

Kidney scarring was assessed using dimercaptosuccinic acid (DMSA) scintigraphy. DMSA scan reports were reviewed to determine the presence and laterality of kidney cortical scarring. Kidney scars were defined as focal or diffuse cortical defects associated with decreased tracer uptake. Based on DMSA findings, patients were categorized into three groups: no kidney scar, unilateral kidney scarring, and bilateral kidney scarring.

### 4.5. Sample Collection and DNA Isolation

Approximately 5 mL of peripheral blood was collected from all participants under aseptic conditions. DNA was extracted using the QIAamp DNA Blood Mini Kit (#51104, Qiagen, Hilden, Germany) according to the manufacturer’s instructions. DNA quality was assessed using a spectrophotometer (NanoDrop One©, Thermo Fisher Scientific, Waltham, MA, USA) with an A260/280 ratio of 1.8–2.0 considered acceptable. Extracted DNA was stored at −80 °C to prevent degradation until the global DNA methylation assay was performed.

### 4.6. Assessment of DNA Methylation

Global DNA methylation levels were quantified using the 5-methylcytosine (5-mC) ELISA Kit (P-1030-96, EpigenTek Group Inc., Farmingdale, NY, USA) according to the manufacturer’s protocol. Briefly, 100 ng of genomic DNA per sample was used. The ELISA quantifies global 5-mC content in genomic DNA using a 5-mC-specific antibody and colorimetric detection, with absorbance measured at 450 nm (Infinite 200 PRO, Tecan Austria GmbH, Grödig, Austria). Methylation was reported as 5-mC%, defined as the percentage of methylated cytosines in the input DNA. The kit-provided negative control DNA (0% 5-mC) and positive control DNA (5% 5-mC) were included on the same 96-well plate alongside participant DNA to generate a standard curve and confirm assay performance.

### 4.7. Statistical Analysis

Data were recorded in Microsoft Excel spreadsheets and analyzed using Stata software, version 14.0. The distribution of continuous variables was assessed using the Shapiro–Wilk test. Data were presented as median (interquartile range) due to the non-normal distribution of methylation levels. The Mann–Whitney U test was used to compare age distribution and 5-mC% between the PUV patients and controls. To quantify distributional separation between groups, Cliff’s Delta was calculated as a non-parametric effect size, expressed as the probability of superiority; because it is rank-based and does not reflect the absolute difference on the 5-mC% scale, we also report median (IQR) and the absolute median difference. Group comparisons of global DNA methylation (5-mC%) across different patient groups (based on CKD stages and scarring status) were performed using the Kruskal–Wallis test, followed by Dunn’s multiple comparisons test for pairwise comparisons. A Bonferroni correction was applied to adjust for multiple comparisons. For exploratory visualization of the relationship between CKD stage and methylation, we plotted the median 5-mC% for each CKD stage and fitted simple trendlines (linear, logarithmic, and exponential); the logarithmic curve provided the highest coefficient of determination (R^2^) and was therefore displayed. This curve-fitting was descriptive only and was not used for hypothesis testing or prediction. Statistical significance was considered at *p* < 0.05. In the box plots, outside values were displayed according to the standard 1.5× IQR rule; no formal outlier test was performed because no data points were excluded from the analyses.

## 5. Conclusions

Compared with controls, boys with PUV demonstrated significantly higher peripheral-blood global 5-mC content. A similar increase in global 5-mC% was observed when PUV-associated CKD subgroups and kidney scarring subgroups were compared with controls. However, no significant differences in global 5-mC% were observed between individual CKD stages or between kidney scarring subgroups. These findings provide preliminary evidence of altered global DNA methylation in PUV and PUV-associated CKD, supporting further evaluation of DNA methylation as a minimally invasive biomarker in this population.

If validated in larger, well-characterized cohorts that include appropriate disease-control groups, methylation profiling may aid risk stratification and longitudinal monitoring in children with PUV. Future studies should also account for potential confounders such as diet and medication exposure and use higher-resolution methylome approaches before definitive conclusions are drawn.

## Figures and Tables

**Table 1:** Clinical characteristics of study participants.

Parameter	PUV Patients
Number of participants	45
Age (months); *median (IQR)*	33 (12–108)
GFR (mL/min/1.73 m^2^); *median (range)*	70 (13–120)
CKD Stage	N = 43
Stage 1 CKD	8 (18.6%)
Stage 2 CKD	19 (44.2%)
Stage 3 CKD	7 (16.2%)
Stage 4 CKD	6 (14%)
Stage 5 CKD	3 (7%)
DMSA Scan	N = 43
No scar	27 (62.8%)
Unilateral scarring	7 (16.3%)
Bilateral scarring	9 (20.9%)
Medications *	
Antihypertensives	6 (13.3%)
Anticholinergics ^#^	22 (48.9%)
Alpha-blockers ^$^	13 (28.9%)

Abbreviations: CKD: chronic kidney disease, DMSA: dimercaptosuccinic acid, GFR: glomerular filtration rate, PUV: posterior urethral valves. * Medications use at the time of sampling. ^#^ Oxybutynin. ^$^ Prazosin.

## Data Availability

The original contributions presented in this study are included in the article/Supplementary Materials. Further inquiries can be directed to the corresponding author.

## Supplementary Materials

The following supporting information can be downloaded at: https://www.mdpi.com/article/10.3390/ijms27135649/s1.

## Abbreviations

The following abbreviations are used in this manuscript:

PUV	Posterior urethral valves
CKD	Chronic kidney disease
5-mC%	5-methylcytosine content
CAKUT	Congenital anomalies of the kidney and urinary tract
DMSA	Dimercaptosuccinic acid
GFR	Glomerular filtration rate
UTI	Urinary tract infections
DTPA	Diethylenetriamine pentaacetic acid
KDIGO	Kidney Disease: Improving Global Outcomes
