# Supplementary material for: Global DNA Methylation in Children with Posterior Urethral Valves: Association with Kidney Function and Kidney Scarring
Source: Int J Mol Sci. 2026 Jun 23;27(13):5649. doi: 10.3390/ijms27135649 (PMC13362500; doi:10.3390/ijms27135649)
Supplement: Supplementary file 1 [file ijms-27-05649-s001.zip › ijms-4352545-supplementary.pdf]

Supplementary Table S1. Clinical and laboratory screening for active urinary tract infection at the time of recruitment

Section	Screening Parameter	PUVs Patients (n = 45)	Controls (n = 45)
**A. Clinical screening**			
	Fever	Absent (0/45)	Absent (0/45)
	Dysuria, increased urinary frequency, or urgency	Absent (0/45)	Absent (0/45)
	Lower abdominal or suprapubic pain	Absent (0/45)	Absent (0/45)
	Flank pain	Absent (0/45)	Absent (0/45)
	Foul-smelling urine	Absent (0/45)	Absent (0/45)
	Vomiting, poor feeding, or lethargy suggestive of infection *	Absent (0/45)	Absent (0/45)
	Abdominal or suprapubic tenderness on examination	Absent (0/45)	Absent (0/45)
	Costovertebral angle tenderness on examination	Absent (0/45)	Absent (0/45)
**B. Laboratory screening**			
	Pyuria on urinalysis	Absent (0/45)	Absent (0/45)
	Bacteriuria on urinalysis	Absent (0/45)	Absent (0/45)
	Positive urine culture (>10^5 CFU/mL)	Absent (0/45)	Absent (0/45)

* Vomiting, poor feeding, and lethargy were assessed particularly in infants and younger children
Abbreviations: PUVs, posterior urethral valves; UTI, urinary tract infection; n, number of participants.
